# Sex-Stratified Skeletal Treatment Effects from the INVEST in Bone Health Randomized Clinical Trial

**DOI:** 10.1093/geroni/igaf122.2495

**Published:** 2025-12-31

**Authors:** Kristen Beavers, Delanie Lynch, Marjorie Howard, Leon Lenchik, Ashley Weaver, Sarah Wherry, Barbara Nicklas, Daniel Beavers

**Affiliations:** Wake Forest University School of Medicine, Winston-Salem, North Carolina, United States; Wake Forest University School of Medicine, Winston-Salem, North Carolina, United States; Wake Forest University School of Medicine, Winston-Salem, North Carolina, United States; Wake Forest University School of Medicine, Winston-Salem, North Carolina, United States; Wake Forest University School of Medicine, Winston-Salem, North Carolina, United States; University of Colorado Anschutz Medical Campus, Aurora, Colorado, United States; Wake Forest University School of Medicine, Winston-Salem, North Carolina, United States; Wake Forest University, Winston Salem, North Carolina, United States

## Abstract

The main goal of the INVEST in Bone Health Trial (NCT04076618) was to compare effects of weight loss (WL) alone (caloric restriction targeting 10% WL), WL plus weighted vest use (WL+VEST; 8 hours/day, weight replacement titrated up to 10% total WL), or WL plus progressive resistance training (WL+RT; 3 supervised sessions/week) on bone health indicators in older adults. As women are more likely to develop osteoporosis and experience fractures, the purpose of this secondary analysis was to present 12-month skeletal treatment effects [including: volumetric bone mineral density (vBMD), areal (a)BMD, and biomarkers of bone turnover (procollagen type 1 N-terminal propeptide (P1NP) and C-terminal cross-linked telopeptide (CTX))] stratified by sex. 150 (74.7% women) older adults (66.4±4.6 years; 68.6% White) living with overweight or obesity (BMI: 33.6±3.3kg/m2) were randomized (n = 50/group) with 133 (89%) completing the trial. Baseline age, weight, height, cardiovascular disease prevalence, and physical activity energy expenditure were higher in men than women. No sex differences were noted in intervention compliance (e.g., weight loss, vest wear-time, RT session attendance). In women, WL+VEST attenuated total hip vBMD loss [estimated treatment difference (ETD): +0.0019 mg/cm3 (0.0005, 0.0034); p = 0.008] and increased P1NP [ETD: +7.6 µg/L (0.8, 14.3); p = 0.014] versus WL alone. WL+RT also increased P1NP versus WL alone [ETD: +5.3 µg/L (-0.3, 10.9); p = 0.028] in women. Conversely, in men WL+VEST exaggerated femoral neck aBMD loss versus WL alone [ETD: -0.038 g/cm2 (-0.066, -0.009); p = 0.012]. In older women, but not men, undergoing caloric restriction, weighted vest use may reduce bone loss via increased bone formation.

